# Population Dynamics of the Mite *Varroa destructor* in Honey Bee (*Apis mellifera*) Colonies in a Temperate Semi-Arid Climate

**DOI:** 10.3390/insects15090696

**Published:** 2024-09-14

**Authors:** Carlos Aurelio Medina-Flores, Alejandro Saucedo Rojas, Ernesto Guzman-Novoa, Luis Alaniz Gutiérrez

**Affiliations:** 1 Unidad Académica de Medicina Veterinaria y Zootecnia, Universidad Autónoma de Zacatecas, Zacatecas 98500, Mexico; alejandro.saucedo.r.95@gmail.com; 2 School of Environmental Sciences, University of Guelph, Guelph, ON N1G 2W1, Canada; eguzman@uoguelph.ca; 3 Facultad de Medicina Veterinaria y Zootecnia No. 2, Universidad Autónoma de Guerrero, Cuajinicuilapa, Guerrero 41940, Mexico

**Keywords:** *Apis mellifera*, *Varroa destructor*, population dynamics, temperate semi-arid climate

## Abstract

**Simple Summary:**

The mite *Varroa destructor* is the most damaging parasite of honey bees (*Apis mellifera*) worldwide. When necessary, mite control is mainly accomplished with acaricides. Optimal parasite control is achieved when the acaricide is applied during times of little or no brood in honey bee colonies, which varies by region. Therefore, in this study we analyzed the population dynamics of the mite in honey bee colonies established in a temperate semi-arid climate in Mexico by periodically sampling brood and adult bees, as well as by counting mites falling to the bottom board of hives over 10 months. We also measured brood and adult bee populations and food stores. It was found that the sampling period influences the population of *V. destructor* in the colonies. The mite population increased by 26% in the 10 months of sampling. It was observed that as the worker brood population increased, the mite infestation rate in adult bees decreased, and the opposite occurred when the amount of brood in the colonies was reduced. Monitoring *V. destructor* populations by recording fallen mites is more reliable than determining mite infestation rates in adult bees and brood. The best period to apply an acaricide treatment in the region of study is between November and December.

**Abstract:**

This study aimed to analyze the population dynamics of the mite *Varroa destructor* in honey bee (*Apis mellifera*) colonies in a temperate semi-arid climate in Mexico. Ten colonies homogeneous in population, food stores, and levels of mite infestation were used. The mite infestation rate in brood and adult bees, total number of mites, daily mite fall, brood and adult bee population, and food stores were determined periodically for 10 months. There was a significant effect (*p* < 0.05) of sampling period on the population of *V. destructor* in adult bees, brood, total mite population, and daily fallen mites. The total mite population increased by 26% on average per colony. The increase in brood amount reduced the mite infestation rate in adult bees, and the opposite occurred when the brood decreased. Monitoring *V. destructor* populations by recording fallen mites is more reliable than determining mite infestation rates in bees, as mite fall has a dynamic pattern similar to that of the total mite population. The best period to apply an acaricide treatment in the region of study is between November and December because most mites were in the phoretic phase, since there was less brood in the colonies compared to other times.

## 1. Introduction

The mite *Varroa destructor* (Acari: Varroidae) [1], is the main health problem for the beekeeping industry worldwide [2]. This is because the mite has had a relatively recent association with *Apis mellifera*, the western honey bee, and has rapidly spread to almost all regions where western honey bee colonies are managed [3,4]. The mite feeds on the fat tissue and hemolymph of the brood and adult bees, inhibiting their immune system and making them more susceptible to bacteria, viruses, fungi, and pesticides [5,6,7,8,9,10]. Additionally, *V. destructor* transmits several viruses to its hosts [11,12,13]. It also shortens the lifespan of parasitized bees [14], reducing the populations and honey yields of their colonies [15,16,17]. For all these reasons, *V. destructor* is one of the main factors associated with the high loss of colonies worldwide [18,19,20].

Besides the damage caused by *V. destructor* to honey bee colonies, the application of acaricides (synthetic and organic) used for its control represents another problem because, in general, all have been shown to have adverse effects on the bees [21,22,23,24,25].

To control mite populations in honey bee colonies and at the same time reduce the use of acaricides, it is necessary to identify the appropriate times to apply treatments. This knowledge would help reduce the negative effects of acaricides on bee health, the selection pressure for mites to develop resistance to the active ingredients of the chemicals, the risk of contamination of hive products, and production costs for beekeepers due to unnecessary or excessive treatment applications [26,27,28,29,30].

Several reports on the population dynamics of *V. destructor* in honey bee colonies from different countries have been published [31,32,33,34,35], but none from Mexico. These reports are valuable for specific countries, but it is critical to take into consideration that the population dynamics of the mite varies regionally due to the seasonality of the brood period in the colonies and its effects on mite reproduction [28,29]. Additionally, very few studies have jointly evaluated the population levels of *V. destructor* in its different life phases (phoretic and reproductive) along with recording daily mite drop and the population and food stores of honey bee colonies.

Studies on the population dynamics of *V. destructor* in honey bee colonies provide information on its evolution and changes in a given region and allow for the establishment of timely control strategies. This study reports for the first time in Mexico the population dynamics of *V. destructor* and considers its phoretic and reproductive phases, as well as natural mite drop, in relation to the population dynamics and food stores of honey bee colonies established in a temperate semi-arid climate.

## 2. Materials and Methods

### 2.1. Study Region

The study was conducted at an experimental apiary belonging to the Veterinary and Animal Science Academic Unit of the Autonomous University of Zacatecas in El Cordovel, General Enrique Estrada, municipality of the state of Zacatecas, Mexico (22°59′42″ N, 102°44′24″ W). This region is characterized by a temperate semi-arid climate, with an average annual precipitation of 400 to 700 mm and an average annual temperature of 18 °C [36].

### 2.2. Experimental Colonies

Ten nucleus colonies homogeneous in bee population, food stores, and *V. destructor* infestation levels were used for the experiments. Six commercial honey bee colonies of mostly European ancestry (previously verified by morphometric analyses) that had not been treated with acaricides for more than two years were used to create the nucleus colonies. The bees from these six colonies were shaken into a large wire cage to blend them. Each nucleus colony was composed of three frames with capped brood and one frame containing honey and pollen. The frames were randomly taken from the six source colonies, while 2 kg of bees were taken from the cage for each nucleus colony [37]. These new colonies were housed in Langstroth hives identified with a number and distributed in a circular arrangement in the apiary separated by 2 m from one another. Three days after establishing the colonies, sister queens of the same age and origin were introduced to the colonies and their presence was monitored during each inspection. The queens were derived by grafting larvae from a colony of European honey bees that was maintained at the research unit for research purposes. The nucleus colonies were established on April 3, 2018—three months before the evaluations began—to ensure that the bee populations studied were daughters of the introduced queens. The colonies were fed weekly with 1.5 L of a 1:1 water/sucrose syrup and 250 g of a commercial food supplement containing 20% protein (Nutra^®^, X-Nox, Aguascalientes, Mexico). Additionally, frames with wax foundation were added as needed by the colonies.

### 2.3. V. destructor Infestation in Adult Bees, Brood, and Daily Mite Fall

The infestation level of *V. destructor* in adult bees and worker brood, as well as the number of mites fallen in the experimental colonies, was determined on five occasions over a 10-month period (3 July, 25 September, and 6 December, 2018, and 28 February and 6 May, 2019).

The mite infestation level in adult bees was determined as per De Jong et al. [38]. This technique involves collecting a sample of approximately 300 adult bees from the brood nest of each colony in a container with 75% ethanol. Subsequently, mechanical agitation was used to remove the mites adhered to the bees. The mite infestation rate was determined by dividing the total number of mites counted by the number of bees analyzed, and the result was multiplied by 100. The infestation level in worker brood was determined by dividing the number of mite-infested cells in a section of capped brood comb (10 × 10 cm) by the number of cells analyzed and multiplying by 100 [39].

With the data on *V. destructor* infestation levels in adult bees and brood, and the population estimates of adult bees and capped brood (described later), the total population of mites in adult bees (phoretic phase) and in the brood (reproductive phase) of the experimental colonies was estimated. This was accomplished by multiplying the average number of mites per bee or brood by the estimated population of adult bees or brood, respectively.

To record fallen mites, a galvanized metallic sheet (28 × 43.5 cm) impregnated with petrolatum was placed on the bottom board of each hive, and a metal mesh (3 mm) of the dimensions of the hive was installed between the sticky sheet and the brood chamber, so that the fallen mites would pass through the mesh and adhere to the sticky sheet. The daily average of fallen mites was obtained by dividing the number of recorded parasites by four, which was the number of days that the adhesive sheets remained in the hives [38].

### 2.4. Bee Population, Brood, and Food Stores of Experimental Colonies

The adult bee population, as well as the comb area containing honey and pollen in the colonies, was calculated by visually estimating the proportion of the surface of each side of combs occupied by these variables. This estimation was performed twice by two different observers, and the values were averaged. To calculate the population of adult bees, the surface proportion occupied by adult bees on each comb was multiplied by 2430, which is the estimated number of adult individuals for a Langstroth brood chamber frame [37]. The proportions for honey and pollen were converted to area (cm^2^) using the surface area of a Langstroth frame on both sides (1760 cm^2^) [37]. The brood population was estimated by determining the area (cm^2^) of capped brood in the colonies. For this purpose, all the combs of each colony were photographed on both sides, and the area in cm^2^ of capped brood in each comb and colony was determined using the ImageJ^®^ software 1.50 National Institute of Health, Bethesda, Maryland, USA). The amount of brood was estimated by multiplying the areas of capped brood by the number of brood cells per cm^2^ (3.9) [37]. Measurements were taken between 6:00 PM and 7:00 PM, when most of the bees were inside the hives. The estimates of the population size of bees, brood, and food stores were also determined every 72 days for 10 months, starting in July 2018.

### 2.5. Statistical Analyses

The data on *V. destructor* infestation rates in brood and adult bees were arcsine-square-root transformed to normalize their distribution. For non-percentage data, normality was verified using the Shapiro–Wilk test. Analysis of variance was used to compare the initial values of *V. destructor* infestation rates, daily mite fall, bee population, and food stores of the experimental colonies. Measures of central tendency and dispersion were obtained for *V. destructor* infestation levels, bee population, and food stores of the colonies. Repeated measures analysis of variance and Tukey tests were also used to compare the effect of sampling time on the measured variables. Additionally, Pearson correlation tests were performed to establish relationships between the evaluated variables. All analyses were conducted using SAS software, version 9.0 [40].

## 3. Results

The colonies did not differ at the beginning of the study in terms of infestation levels in adult bees (F_1,9_ = 2.51, *p* = 0.35), brood (F_1,9_ = 2.42, *p* = 0.36), and daily fall of *V. destructor* (F_1,9_ = 3.0, *p* = 0.33). There were also no differences between colonies in initial bee population (F_1,9_ = 1.72, *p* = 0.41), amount of brood (F_1,9_ = 0.81, *p* = 0.53), and areas of pollen (F_1,9_ = 0.48, *p* = 0.61) and honey (F_1,9_ = 1.69, *p* = 0.42).

During the course of the study, significant differences were found between sampling periods for *V. destructor* infestation rates in adult bees (F_4,46_ = 4.08, *p* = 0.008) and brood (F_4,46_ = 11.82, *p* < 0.0001), as well as for the number of fallen mites (F_4,46_ = 7.81, *p* = 0.0001), amount of brood (F_4,46_ = 39.27, *p* < 0.0001), adult bee population (F_4,46_ = 13.33, *p* < 0.0001), mite population in brood (F_4,46_ = 10.33, *p* < 0.0001), mite population in adult bees (F_4,46_ = 5.36, *p* = 0.001), total mite population (F_4,46_ = 13.51, *p* < 0.0001), areas of pollen (F_4,46_ = 15.19, *p* < 0.0001), and areas of honey (F_4,46_ = 25.59, *p* < 0.0001).

**Figure 1:**
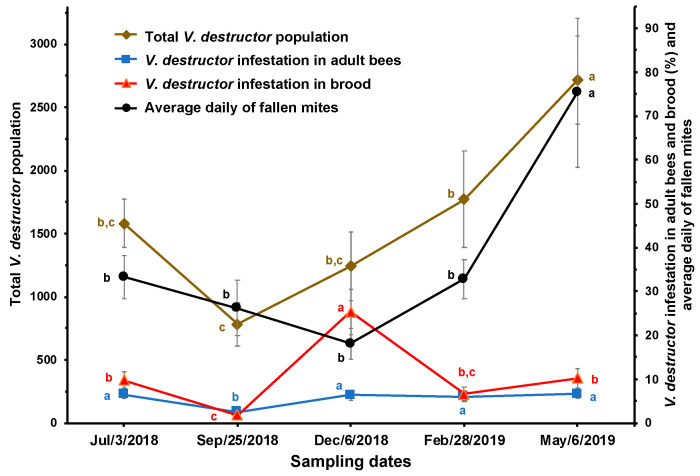
Estimated total population of *V. destructor*, brood and adult bee infestation (%), and average daily mite fall (±SE) at different times of the year in honey bee colonies (*n* = 10) established in a temperate semi-arid climate. Different letters between means for each variable indicate significant differences based on analyses of variance and Tukey tests.

In September, the colonies had the lowest infestation rates in brood and adult bees and the lowest number of estimated total mites, while in December, the colonies had the highest mite infestation rate in the brood. In May, the colonies had the highest number of mites fallen onto the adhesive sheets and the highest estimated number of total mites in the colonies (Figure 1).

**Figure 2:**
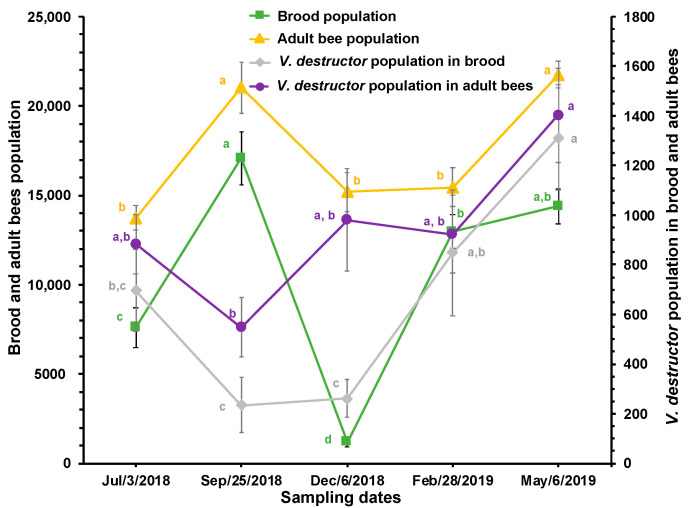
Estimated population of brood, adult bees, and *V. destructor* in brood and adult bees (±SE) at different times of the year in honey bee colonies (*n* = 10) established in a temperate semi-arid climate. Different letters between means for each variable indicate significant differences based on analyses of variance and Tukey tests.

Figure 2 shows that there was a greater population of adult bees and brood during the months of September and May, while the estimated number of *V. destructor* mites on adult bees showed significant differences only between September and May. Additionally, the estimated number of mites in the brood was significantly higher in May compared to the period between the previous June and December. Moreover, a reduction in the population of *V. destructor* in adult bees was observed in response to an increase in the amount of brood in the colonies, and an increase in the population of *V. destructor* in adult bees was observed when the amount of brood in the colonies decreased. The lowest estimated number of *V. destructor* mites in the brood compared to the number of mites on adult bees was also observed in December.

**Figure 3:**
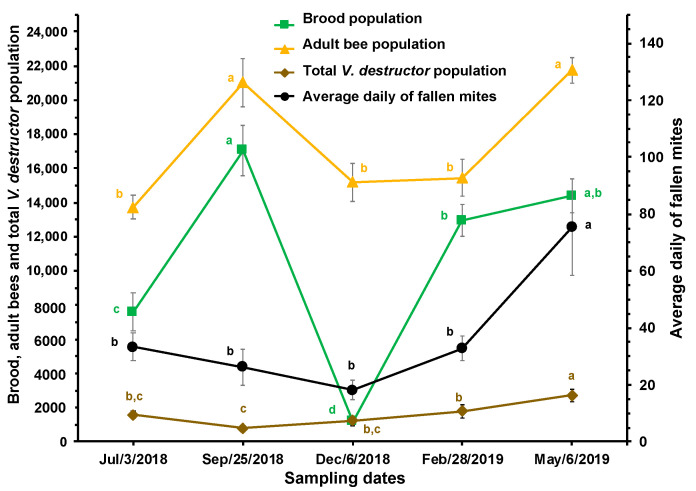
Estimated brood, adult bees, and total *V. destructor* populations and average daily mite drop (±SE) at different times of the year in honey bee colonies (*n* = 10) established in a temperate semi-arid climate. Different letters between means for each variable indicate significant differences based on analyses of variance and Tukey tests.

A decrease (although not significant) in the estimated total number of *V. destructor* mites (including mites in both the reproductive and phoretic phases) relative to the populations of brood and adult bees, which increased significantly, was observed from July to September. The estimated total population of *V. destructor* in the colonies was lowest in September, with an average of 782 ± 168 mites, a number that progressively and significantly increased to reach 2715 ± 350 mites in May. Following a similar pattern, the daily average of fallen mites showed no significant differences from July to February, with an average of 27.6 ± 1.6 parasites, while the mean of that variable was significantly higher in May (75.3 ± 5.6; Figure 3).

**Figure 4:**
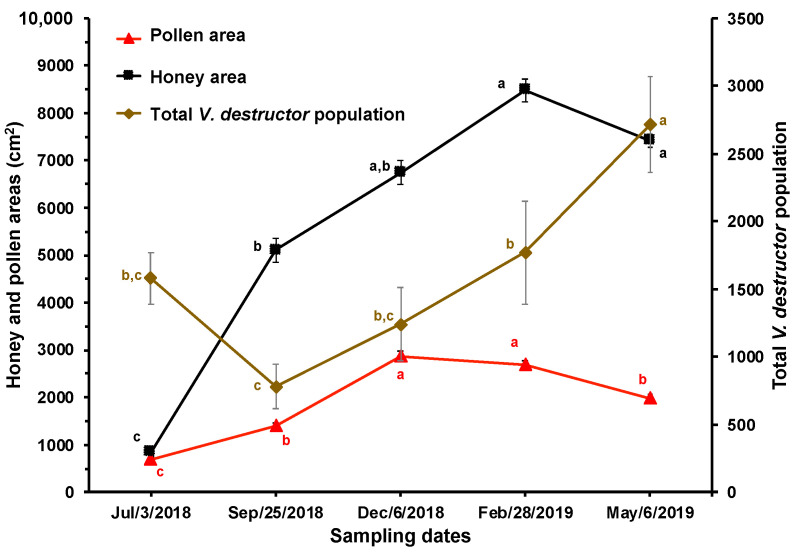
Honey and pollen areas (cm^2^) and estimated total population of *V. destructor* mites (±S.E.) at different times of the year in honey bee colonies (*n* = 10) established in a temperate semi-arid climate. Different letters between means for each variable indicate significant differences based on analyses of variance and Tukey tests.

From September through February, there was a parallel and significant increase in the total population of *V. destructor* and the honey and pollen stores in the brood chambers of the experimental colonies (Figure 4).

Significant correlations were found between the rates of *V. destructor* infestation (in adult bees and brood) and daily mite drop, total *V. destructor* population, and amount of brood. The daily mite drop was related to the infestation rate in adult bees. Additionally, the amount of brood was also related to the adult bee population, while the honey areas were significantly correlated with the pollen areas of the colonies (Table 1).

## 4. Discussion

The differences found in the estimated populations of *V. destructor* in brood, adult bees, and total mites, as well as in the number of parasites fallen between the analyzed time periods, are likely due to changes in environmental conditions and the availability of food resources for the colonies over the 10 months monitored. These factors are known to influence variations in the queen’s egg-laying rate [28,29,41] and consequently, in the availability of brood for the reproduction of *V. destructor.*

There is a close relationship between the population dynamics of the parasite and its host, which is apparently confirmed by the reduction in mite infestation rate in brood and adult bees between July and September. This appears to be due to the increase in the brood and worker bee populations observed during that period, causing a dilution effect on the mite infestation rates. The increase in the amount of brood encouraged many mites in the phoretic phase to leave the adult bees to enter cells containing larvae to reproduce. Conversely, the increase in the infestation rate of *V. destructor* in adult bees in December is likely a consequence of mite reproduction between July and September and a reduction in the bee and brood populations (see Figure 1 and Figure 2). From September to January, the amount of brood decreased, thereby reducing the possibilities for mite reproduction and population growth. From February to May (spring blooming season), the amount of brood increased, leading to a significant multiplication of *V. destructor*, probably due to the greater availability of food resources for the colonies during that time of year, resulting in greater production and availability of larvae for the mite to reproduce. Although there was no significant correlation between the *V. destructor* population and the stores of honey and pollen, Figure 4 shows an increase in the mite population when food stores increased. In September and October, as well as between February and May, there are many plants blooming in the study area, which favored colony development and the storage of honey and pollen in the combs. It has been reported that the amount of pollen in the colonies is related to the fertility of the mite [42].

The results of this study show that when the amount of brood increases (between July and September), the mite infestation rate in adult bees decreases because many parasites migrate from the adult bees to the brood. Inversely, when the amount of brood decreases (September to December), the number of mites and the infestation rate in adult bees increase because there are not enough larvae for the parasites to reproduce [35]. 

The estimated total mite population tended to decrease when the bee population in the colonies increased (see Figure 3). This is possibly because before July there was little brood available for the parasite to reproduce, and this is simply a delayed effect of the population dynamics, resulting in a decrease in mite population due to low reproduction during the period of little brood before July. However, from September onward, there was a progressive increase in the mite population.

The results presented in Figure 1 and Figure 3 show that monitoring *V. destructor* populations by recording fallen mites is more reliable than determining mite infestation rates in bees, as mite fall has a dynamic pattern similar to that of the estimated total mite population. Additionally, the techniques for determining infestation levels in brood and adult bees are destructive, negatively impacting the colony population, particularly when infestation levels are determined frequently [43]. This conclusion is reinforced by the correlation found between daily mite drop and the estimated total mite population in the colonies. Overall, the results confirm that mite fall is a relatively effective technique for determining *V. destructor* infestation levels in colonies.

Contrary to what was reported by Branco et al. [43], our results suggest that the rate of *V. destructor* in adult bees and brood is an imprecise and uninformative measure for estimating the total mite population in the colonies. This is due to the migration of *V. destructor* during the phoretic and reproductive phases of the mite, and the fact that the population dynamics of the parasite are related to the cycle and amount of brood and adult bees in the colonies, which vary across different seasons of the year [28,29]. The increase in the brood and adult bee population in the colonies reduces the proportion of mites relative to the bee population, while a decrease in the colony population leads to a higher mite infestation rate [44]. Additionally, when the amount of brood decreases and the number of adult bees increases, the percentage of mite infestation in adult bees rises, as occurred during December; this is the time of year with the least brood area in the colonies, which causes most mites to remain in the phoretic phase, concentrating the *V. destructor* population on adult bees (see Figure 2).

For colonies with *Varroa* infestation levels exceeding the treatment threshold, the period from November to December is optimal for applying an acaricide, as most of these chemical products do not affect mites inside capped brood cells but do affect them in the phoretic phase. Thus, during these months (at the end of the fall honey harvest), there is a window of opportunity to apply an acaricide treatment in regions with a temperate semi-arid climate of the Mexican highlands. Specifically, this is the time of year when products such as oxalic acid are most effective [45]. Additionally, it is important to consider that acaricide treatment during this period will limit *V. destructor* reproduction before the expected increase in bee population in the early part of the year (starting in February). It is also important to consider that the treatment threshold suggested for Mexico by SAGARPA [46] is 5% infestation in adult bees and/or 10 mites fallen in 24 h, which aligns with the results of a study conducted in the central highlands region of Mexico, where it was found that infestation levels of 4.5% in adult bees do not significantly affect population parameters, food stores, or colony weight [47]. Proper and timely application of a treatment helps reduce the negative effects of acaricides on bees and brood, as well as the selection pressure for resistant mites, the risks of contamination of hive products, and the production cost for beekeepers due to unnecessary treatment applications; it also prevents selection pressure from relaxing to develop mite-resistant honey bee colonies [26,27,28,29,30].

In a study conducted with Africanized honey bee colonies in the Mexican tropics, it was found that the population of *V. destructor* decreased by more than 1000 mites over twelve months. This was attributed to the low production of fertile mites in each reproductive cycle of *V. destructor* (0.7) during a time of year with high worker larvae mortality [48]. Conversely, in the present study conducted in a temperate semi-arid region of Mexico using colonies of bees with greater European ancestry, an increase of 1136 mites per colony on average over ten months was observed, representing a 26% increase in the total mite population. The record of mites fallen onto the adhesive boards of the hives showed a 39% increase in final infestation levels compared to the initial levels (33 ± 1.5 mites in July vs. 75.3 ± 5.6 in May). The increase in mite population observed in this study is similar to that reported in honey bee colonies in Costa Rica [33]. The population size of drone brood in the colonies and the low expression of bee resistance mechanisms against the mite may have been factors that partly explain the *V. destructor* population increase in this study, although many more factors not studied here could have contributed to the mite’s population growth and its temporal variations.

Jack and Ellis [49] developed a formula to estimate the overall population of *V. destructor* in a honey bee colony based on the number of mites naturally fallen onto an adhesive board installed in a hive. We found that our total estimate of mites/hive was on average lower than the number of mites predicted by the formula. This is probably due to the fact that in our study we did not consider the population of *V. destructor* in drone brood.

The population dynamics of *V. destructor* are variable and depend on multiple factors and their interactions [45,50]. The main factors that determine the population growth rate of *V. destructor* and its pathogenicity in honey bee colonies are its reproductive ability and longevity [45,51], as well as the climatic conditions and nectar flow that influence the availability of brood and drones, the generation of swarms [28,29,41], management practices [50], and the overcrowding of colonies, which can favor bee drift and robbing [52,53,54]. Other factors include the genotype of the colonies [55,56,57] and the expression of bee defense mechanisms that limit the reproduction and survival of the mite. The mechanisms of resistance that most restrain the population growth of *V. destructor* in honey bee populations of Latin America include hygienic behavior, grooming behavior, low brood attractiveness, suppression of mite reproduction, and other mite non-reproduction related mechanisms that result in low fertility and fecundity of the mite [58,59].

Studies have reported variable results regarding the correlations between colony population parameters and *V. destructor* infestation levels, as well as correlations between infestation levels measured by different methods [27,31,33,42,43,48,55]. Therefore, there is no consensus on the effect of these parameters on the population dynamics of the mite. Moreover, complex multifactorial interactions exist that make it difficult to accurately predict mite populations in colonies [45]. The results of this study provide new insights into the population dynamics of *V. destructor* in a temperate semi-arid climate region and suggest that more studies are needed to identify the limiting factors of mite reproduction, as well as the seasonality of mite population dynamics in different regions and climates. This knowledge will help establish sustainable control strategies for this harmful parasite of honey bees.

## 5. Conclusions

The results of this study demonstrate a close relationship between the population dynamics of *V. destructor* and that of honey bee colonies. In December, there was an increase in the number of mites infesting adult bees, as well as a decrease in the number of mites infesting brood. Therefore, it is advisable to apply an acaricide treatment during this period. Another significant finding of this study is that relying on mite drop data for estimating the *V. destructor* infestation levels of honey bee colonies provides a more reliable indication of the mite population in the colonies than data on brood and adult bee infestation rates. Similar studies are needed for other regions and climates, as well as studies that relate climatic variables to the population dynamics of *V. destructor* in order to generate knowledge that will enable the development of effective and sustainable mite control strategies.

## Figures and Tables

**Table 1:** Correlation coefficients between *V. destructor* infestation rates in brood and adult bees, daily mite drop, total *V. destructor* population, adult bee population, amount of brood, and areas of honey and pollen in honey bee colonies (*n* = 10) established in a temperate semi-arid climate.

Variable	Infestation Adult	Infestation Brood	Fallen Mites	Total Mites	Brood Amount	Adult Population	Pollen Area
Infestation brood	0.48 ***						
Fallen mites	0.43 **	0.12 ^ns^					
Total mites	0.73 ***	0.40 **	0.62 ***				
Brood amount	−0.30 *	−0.65 ***	0.13 ^ns^	0.04 ^ns^			
Adult population	−0.23 ^ns^	−0.18 ^ns^	0.17 ^ns^	0.15 ^ns^	0.59 ***		
Pollen area	0.20 ^ns^	0.19 ^ns^	0.18 ^ns^	0.10 ^ns^	−0.12 ^ns^	−0.02 ^ns^	
Honey area	0.17 ^ns^	0.18 ^ns^	0.10 ^ns^	0.30 ^ns^	0.15 ^ns^	0.21 ^ns^	0.45 ***

***, *p* < 0.001; **, *p* < 0.01; *, *p* < 0.05; ^ns^ = Not significant. The correlation coefficients were obtained using Pearson’s test.

## Data Availability

All data are available in this paper.
